# Supplementary material for: Altered microRNA Transcriptome in Cultured Human Liver Cells upon Infection with Ebola Virus
Source: Int J Mol Sci. 2021 Apr 6;22(7):3792. doi: 10.3390/ijms22073792 (PMC8038836; doi:10.3390/ijms22073792)
Supplement: Supplementary file 1 [file ijms-22-03792-s001.zip › Supplementary_File/C_ GO_Analysis_Results/16-30nt_go_Mayinga-96h-Huh7_vs_Control-96h-Huh7_up.mature_mirna_targets/BP_EnrichmentScoreDotPlot.pdf]

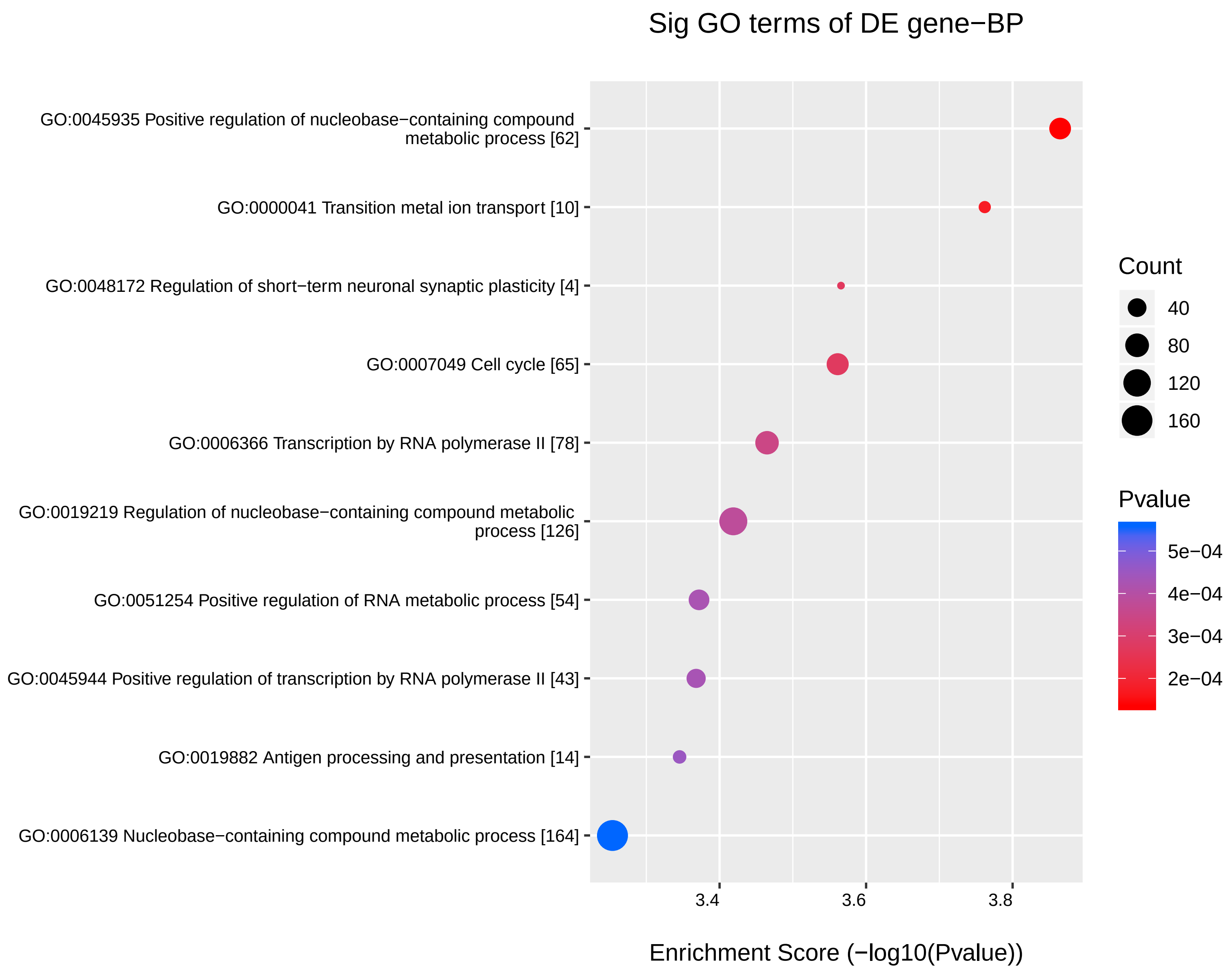
Sig GO terms of DE gene−BP
GO:0045935 Positive regulation of nucleobase−containing compound metabolic process [62]
GO:0000041 Transition metal ion transport [10]
GO:0048172 Regulation of short−term neuronal synaptic plasticity [4]
GO:0007049 Cell cycle [65]
GO:0006366 Transcription by RNA polymerase II [78]
GO:0019219 Regulation of nucleobase−containing compound metabolic process [126]
GO:0051254 Positive regulation of RNA metabolic process [54]
GO:0045944 Positive regulation of transcription by RNA polymerase II [43]
GO:0019882 Antigen processing and presentation [14]
GO:0006139 Nucleobase−containing compound metabolic process [164]
3.4
3.6
3.8
Enrichment Score (−log10(Pvalue))
Count
40
80
120
160
Pvalue
5e−04
4e−04
3e−04
2e−04
